# QPSO-MPC based tracking algorithm for cable-driven continuum robots

**DOI:** 10.3389/fnbot.2022.1014163

**Published:** 2022-10-14

**Authors:** Qi Chen, Yanan Qin, Gelun Li

**Affiliations:** ^1^School of Mechanical Engineering, University of Shanghai for Science and Technology, Shanghai, China; ^2^Robotics State Key Laboratory, Shenyang Institute of Automation, Chinese Academy of Sciences, Shenyang, China

**Keywords:** cable-driven continuum robots, QPSO, MPC, control stability, trajectory tracking precision

## Abstract

Cable-driven continuum robots (CDCRs) can flexibly travel through narrow space for complex workspace tasks. However, it is challenging to design the trajectory tracking algorithm for CDCRs due to their nonlinear dynamic behaviors and cable hysteresis characteristics. In this contribution, a model predictive control (MPC) tracking algorithm based on quantum particle swarm optimization (QPSO) is designed for CDCRs to realize effective trajectory tracking under constraints. In order to make kinematic analysis of a CDCR, the forward and inverse mapping among actuation space, joint space and work space is analyzed by using the piecewise constant curvature method and the homogeneous coordinate transformation. To improve the performance of conventional MPC for complex tracking tasks, QPSO is adopted in the rolling optimization of MPC for its global optimization performance, robustness and fast convergence. Both simulation and operational experiment results demonstrate that the designed QPSO-MPC presents high control stability and trajectory tracking precision. Compared with MPC and particle swarm optimization (PSO) based MPC, the tracking error of QPSO-MPC is reduced by at least 43 and 24%, respectively.

## Introduction

The continuum robots, designed by imitating the biological characteristics of snakes and elephant trunks, have multiple degrees of freedom (DOFs) (Guo et al., 2018; Guan et al., 2020). In comparison with conventional industrial robots, continuum robots are more suitable for working in the uncertain environment due to their deformable structures. The actuation mechanism of continuum robots includes flexible fluidic actuations (FFAs) (Garriga-Casanovas et al., 2018; Renda et al., 2020), shape memory alloy materials (SMAs) (Yang et al., 2018; Jiang et al., 2020), electroactive polymers (EAPs) (Chang et al., 2018; Bar-Cohen and Anderson, 2019) and cable-driven actuations (CDAs) (Jin et al., 2018; Hamida et al., 2021). Cable-driven continuum robots (CDCRs) are activated by changing link lengths, which make them easy to operate (Khomami and Najafi, 2021). Compared with other continuum robots, CDCRs have many advantages such as light weight, small moment of inertia and easy to realize variable stiffness control. CDCRs are supported by passive joints and elastic backbones. By changing the lengths of the driving cables, CDCRs are able to achieve bending, stretching, twisting, grasping and other actions. Therefore, CDCRs are widely used in rescue operations (Kim et al., 2020; Takahashi et al., 2021), minimally invasive surgery (Miyasaka et al., 2015; Hwang et al., 2020), rehabilitation applications (Zhang et al., 2020; Shi et al., 2021) and human–robot interaction (Ma et al., 2021a,b). The trajectory tracking is essential for CDCRs to realize various tasks. However, it is challenging for CDCRs to track the desired trajectory accurately and smoothly due to their nonlinear response, parameters variation and non-rigid structures.

In recent years, a series of researches have emerged in motion control and trajectory tracking of CDCRs. A bio-inspired CDCR was designed in Li and Du (2013), where the kinematic model derived from the piecewise constant curvature method was used to control the motion of the robot. A segmented constant curvature method was employed to derive the forward and inverse kinematic model of a CDCR, which achieved simple shape and posture control of the CDCR (Liu et al., 2021). The kinematic model based controllers are well-understood and easy to apply. However, uncertainties incurred in exact kinematic calculations make kinematic-based tracking controllers suffer from accumulative errors in performing complex trajectory tracking tasks.

Researchers have developed adaptive trajectory tracking methods to solve the problem of modeling errors. Wang et al. (2020) designed an adaptive time-delay approach for the trajectory tracking of CDCRs, which enhances robustness of the controller. Li et al. (2020) introduced a pretension-based adaptive robust controller, which solved the problem of the cable slack in the process of trajectory tracking. However, the performance of adaptive controllers depends on a computational expensive process of updating the controller parameters.

Neural network control method is also used to achieve trajectory tracking by previous training samples. Tan et al. (2021) adopted neurodynamics method to track the preset trajectory of a CDCR. The end position control of a CDCR was implemented based on deep reinforcement learning in Wu et al. (2020). Since the CDCR has a nonlinear structure with continuous bending and infinite DOFs, the training samples are difficult to obtain and the huge amount of computation cannot meet the demand of complex tasks.

Researchers have also focused on sliding mode control (SMC) for its simple structure. Liu and Xia (2020) introduced a trajectory tracking control system based on SMC for a three DOFs CDCR. Abu et al. (2019) designed a multi-surface SMC to solve the nonlinear problem of CDCRs. However, sliding mode controllers produce buffeting due to the cable-related effects.

Fuzzy control calculates tracking strategy from expert knowledge, which has a strong anti-interference ability. Ba et al. (2021) implemented a fuzzy-logic feedback controller to track evenly distributed points. Compared with the piecewise constant curvature (PCC) based controller, the proposed controller provides a solution to the problem of failing to converge. A closed-loop fuzzy PID controller was proposed for position control of a CDCR in Xu et al. (2018). However, the fuzzy rules are subjective and difficult to obtain in practice due to the complex structure of CDCRs.

The shape-changing structures of CDCRs result in control constraints, which make it difficult to control CDCRs to follow the desired trajectory. Moreover, the error of state estimation and external interference can also affect the tracking precision. This makes conventional control methods difficult to achieve stable trajectory tracking for complex tasks. MPC is widely used for trajectory tracking tasks thanks to its ability of dealing with various constraints (Chu et al., 2021). The rolling optimization is essential for MPC based controllers, which can compensate for model uncertainties and disturbances. However, the nonlinear and time-varying dynamic characteristics of CDCRs make the rolling optimization design of MPC challenging. A nonlinear MPC based on the particle swarm optimization (PSO) was designed for a CDCR to track the expected trajectory (Amouri et al., 2022). However, PSO cannot ensure global optimal outputs due to the limitation of the search space of particles. The quantum particle swarm optimization (QPSO) cancels the particle moving direction attribute of PSO to increase the randomness of particle motion, so the update of particle positions is no longer constrained by the previous motion state and the problem of local minima is also alleviated. A QPSO based path optimization approach was used to achieve a smooth trajectory in a complex plane with obstacles, which can avoid local minimum and enhance the probability of searching global optimal control points (Dian et al., 2022). In this paper, a QPSO based MPC is designed to the trajectory tracking control for CDCRs, where the QPSO is used in the rolling optimization process of MPC. Compared with the traditional rolling optimization process of MPC, the QPSO algorithm adopts the average optimal position to improve the cooperation between particles, which can improve global search performance significantly. The proposed MPC controller achieves global optimal performance, robustness and fast convergence. The contribution of our work is that the proposed QPSO algorithm provides optimal control outputs of MPC to compensate the uncertainties caused by model mismatch, distortion and interference. Simulations and experiments show that the QPSO-MPC trajectory tracking controller has high tracking stability and accuracy in complex tasks.

This paper is arranged as follows: Section Modeling of the CDCR designs the structure and model of the CDCR. The QPSO-MPC trajectory tracking controller is designed in section QPSO-MPC trajectory tracking strategy. Section Simulation results presents the simulation and analysis results in detail. In Section Experiment results, three typical experiments are made and discussed. Finally, Section Conclusions draws conclusions.

## Modeling of the CDCR

### Mechanical structure of the CDCR

**Figure 1:**
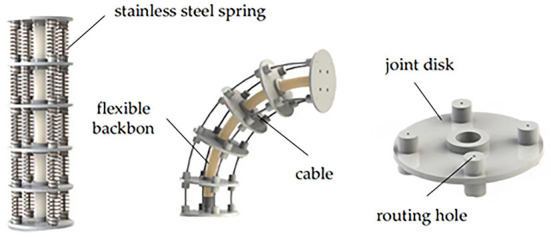
The structure of the CDCR.

The structure of the designed CDCR is shown in Figure 1. The robot is composed of 6 joint disks (diameter, 4 cm; distance between joint disks, 6 cm) and a flexible backbone made of silicone rubber (diameter, 1 cm; length, 30 cm). Four high-strength polyethylene fiber cables are evenly distributed at an interval of 90° along the length of the backbone. Each cable is, respectively, attached to a servo driver and terminated at the last joint disk. The cables are pulled by the drivers to make the robot bend at a particular angle.

### Kinematic model of the CDCR

It is difficult to derive the kinematic model of the under-actuated CDCR owing to its complex structure. Before analyzing the kinematic model of the robot, the following key assumptions need to be made and emphasized:

(1) Assume that the robot bends with a constant curvature.
(2) Assume that the flexible backbone of the robot is incompressible.
(3) The robot is driven by four evenly distributed traction cables that are also incompressible.

The CDCR is not a kind of direct driving robot, which is controlled by the length changes of the cables. The actuation space ***L*** is denoted as the length changes of the cables. The joint space **Θ** is the set of the bending angle and the rotation angle, which is controlled by the length changes of the cables. The work space ***W*** represents the position of the end joint disk, which can be obtained from the joint space. Hence, the kinematic models of the robot can be derived from the mapping relationship among ***L*** , **Θ** and ***W*** .

**Figure 2:**
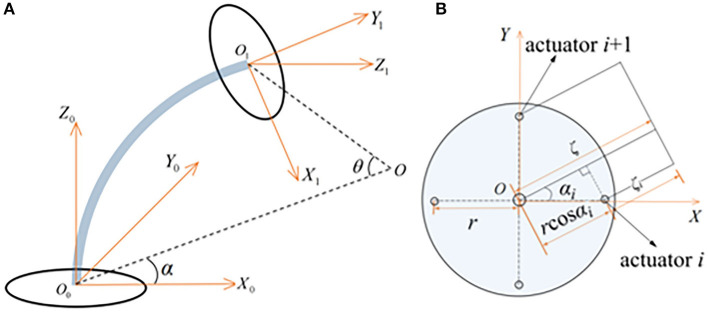
Kinematic model of the CDCR: **(A)** Single joint bending geometric; **(B)** Vertical view of the base section.

The single joint geometric model of the CDCR is shown in Figure 2A. The routing holes of the 4 driving cables are marked from 1 to 4. The base frame and end-effector frame of the CDCR are established in the center of the base joint and the end joint disk, respectively. The robot has a bending DOF and a rotation DOF, which are expressed by bending angle θ and rotation angle α, respectively. *O* is denoted as the center of the bending curve. α is the intersection angle between plane *X*_0_*Z*_0_*O*_0_ and plane *OO*_0_*O*_1_, and θ is the center angle of the curvature arc.

Figure 2B provides a vertical view of the base section. Under the premise of the assumption of constant curvature, the radius of curvature for each driving cable is expressed as follows:


(1) $$\zeta {\;}_{\text{i}}\text{=}\zeta \text{ -rcos}\alpha {\;}_{\text{i}}$$

where ζ stands for the radius of curvature measured from the center of the disk, ζ_*i*_ stands for the bending curvature radius of the *i*-th cable (*i* = 1, 2, 3, 4), *r* is the distance between the center of the disk and the center of each routing hole, and the rotation angle of the *i*-th cable is denoted as α_*i*_= α - (*i*- 1) π/2.

The 4 driving cables are parallel to each other. Multiplying the bending angle θ with Equation (1), the length change of the driving cables is derived as follows:


(2) $$\begin{array}{l} \text{ Δ }l_{i}=\text{(}\zeta \text{-}{\zeta \;}_{\text{i}}\text{)}\theta \text{=r }\theta \;\text{cos}\alpha_{i} \end{array}$$

where Δ*l*_*i*_ represents the length change of the *i-*th driving cable. The relationship between joint space and work space can be derived by applying Equation (2) to the *i*-th and the *i*+1-th actuators and getting the quotient of them. Since α__*i*_+1_ = α_*i*_ - *i*π/2, the joint space variables are obtained as follows:


(3) $$\begin{array}{l} \text{α}=\text{arctan(}\frac{\text{ Δ }l_{i+\text{1}}}{\text{ Δ }l_{i}}\text{)} \end{array}$$

(4) $$\begin{array}{l} \theta =\frac{\text{ Δ }l_{i}}{r\text{cosα}} \end{array}$$

The relative pose from the base frame to the end-effector frame is represented by the transformation matrix ***T*** ∈ R^4 × 4^, which is composed of the rotation matrix ***R*** ∈ R^3 × 3^ and the position vector ***Q*** ∈ R^3^. Based on the geometric analysis method, the rotation matrix ***R*** is given by the consecutive rotations about the *Z* and *Y* axis as follows:


(5) $$\begin{array}{l} \text{R}=\left[\begin{array}{ccc} {\text{c}}^{2}\alpha \text{c}\theta +{\text{s}}^{2}\alpha & \text{c}\alpha \text{s}\alpha \text{c}\theta -\text{c}\alpha \text{s}\alpha & \text{c}\alpha \text{s}\theta \\ \text{c}\alpha \text{s}\alpha \text{c}\theta -\text{c}\alpha \text{s}\alpha & {\text{s}}^{2}\alpha \text{c}\theta +{\text{c}}^{2}\alpha & \text{s}\alpha \text{s}\theta \\ -\text{c}\alpha \text{s}\theta & -\text{s}\alpha \text{s}\theta & \text{c}\theta \end{array}\right] \end{array}$$

where c and s represent cos and sin, respectively. The position vector ***Q*** is expressed as follows:


(6) $$\begin{array}{l} Q={\left[\begin{array}{ccc} \frac{l}{\theta }\text{c}\alpha \left(1-\text{c}\theta \right) & \frac{l}{\theta }\text{s}\alpha \left(1-\text{c}\theta \right) & \frac{l}{\theta }\text{s}\theta \end{array}\right]}^{\text{T}} \end{array}$$

Then the pose transformation matrix ***T*** is expressed as follows:


(7) $$\begin{array}{l} \text{T}=\left[\begin{array}{cc} \text{R} & Q\\ 0 & \text{1} \end{array}\right] \end{array}$$

The relationship between joint space and work space can be derived by the transformation matrix ***T*** . In order to solve the inverse kinematic transformation from work space to joint space, the end-effector transformation matrix ***T*** is presented as follows:


(8) $$\begin{array}{l} \text{T}=\left[\begin{array}{cccc} w_{x} & e_{x} & b_{x} & q_{x}\\ w_{y} & e_{y} & b_{y} & q_{y}\\ w_{z} & e_{z} & b_{z} & q_{z}\\ 0 & 0 & 0 & 1 \end{array}\right] \end{array}$$

where ***w*** , ***e*** , ***b*** are the unit vectors corresponding to the *X*_1_, *Y*_1_, *Z*_1_ axes, respectively, and ***Q*** is the position vector. The bending angle θ and the rotation angle α of the robot is given by Equation (9)-(10):


(9) $$\begin{array}{l} \theta =\text{arccos(}b_{\text{z}}\text{)} \end{array}$$

(10) $$\begin{array}{l} \alpha =\arctan\left(\frac{q_{y}}{q_{x}}\right) \end{array}$$

## QPSO-MPC trajectory tracking strategy

**Figure 3:**
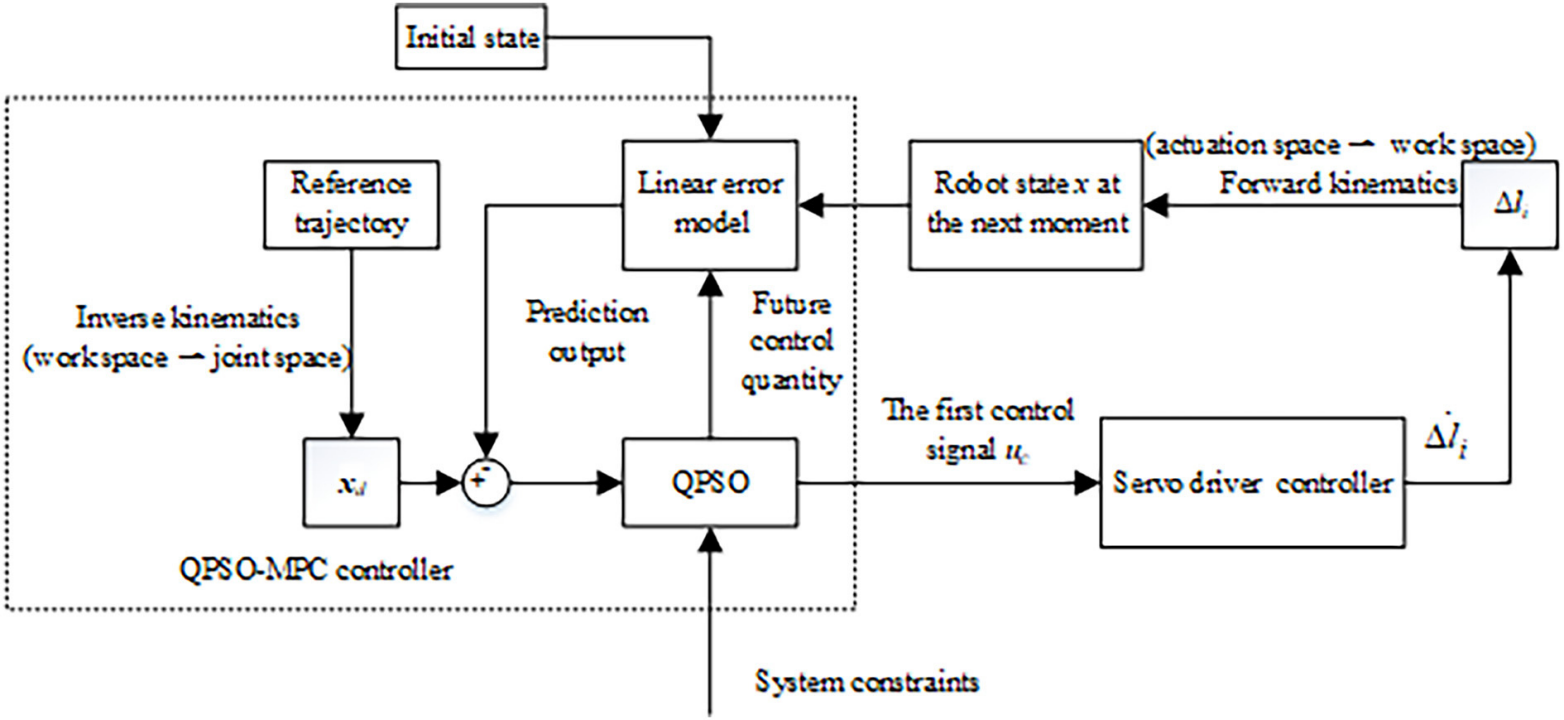
The block diagram of the trajectory tracking controller based on QPSO-MPC.

The rolling optimization process of MPC is essential because it can repeatedly produce online control signals to compensate system uncertainties. To reduce the tracking error of MPC, QPSO is adopted in the rolling optimization process to improve tracking stability and accuracy. The block diagram of the designed tracking algorithm is shown in Figure 3, which is mainly composed of system constraints, the error model and the optimization process based on QPSO. According to the inverse kinematic equation from work space to joint space, the reference trajectory and the real trajectory are transformed into the joint space as input to the QPSO-MPC controller. The linear error model predicts the future position of the CDCR through mathematical description. The optimization process based on QPSO makes the CDCR has high control stability and tracking precision. Compared with the rolling optimization process of MPC, QPSO ensures an optimal feasible control value at every moment, which makes the robot track the target trajectory more stably and accurately.

### Linear error model

The configuration state vector of the control system is ***s*** = [*x, y, z*, θ, α]^T^, and the control vector is ***h*** = [$\overset{•}{\theta },\overset{•}{\alpha }$]^T^. The state of the control system can be expressed as follows:


(11) $$\begin{array}{l} ṡ=\text{f}\left(s,h\right) \end{array}$$

The velocity kinematics of the end-effector is described by taking the Jacobian matrix of the work space with respect to joint space. By taking the derivative of Equation (6), the velocity of the end-effector $\overset{•}{V}={\left[ẋ,ẏ,ż\right]}^{\text{T}}$is obtained as follows:


(12) $$\begin{array}{l} \overset{•}{V}=J\left(\text{Θ}\right)\overset{•}{\text{Θ}}=\left[\begin{array}{cc} -l\text{c}\alpha \frac{1-\text{c}\theta -\text{s}\theta \theta }{\theta^{2}} & -l\text{s}\alpha \frac{1-\text{c}\theta }{\theta }\\ -l\text{s}\alpha \frac{1-\text{s}\theta \theta -\text{c}\theta }{\theta^{2}} & -l\text{c}\alpha \frac{\text{c}\theta -1}{\theta }\\ -l\frac{\text{c}\theta \theta -\text{s}\theta }{\theta^{2}} & 0 \end{array}\right]\left[\begin{array}{c} \overset{•}{\theta }\\ \overset{•}{\alpha } \end{array}\right] \end{array}$$

where ***J*** (**Θ**)∈***R*** ^3 × 2^ is the Jacobian matrix. According to Equation (12), the state space of the system is expressed as follows:


(13) $$\begin{array}{l} \left[\begin{array}{c} \overset{•}{V}\\ \overset{•}{\text{Θ}} \end{array}\right]=\left[\begin{array}{c} \text{J}\left(\text{Θ}\right)\overset{•}{\text{Θ}}\\ \overset{•}{\text{Θ}} \end{array}\right] \end{array}$$

By performing Taylor series expansion of Equation (11) at the reference trajectory ***s***_*d*_ and ignoring the higher order terms (11) can be defined as follows:


(14) $$\dot{\text{s}}=\text{f}({\text{s}}_{d},{\text{h}}_{d})+\frac{\delta \text{f}}{\delta \text{s}}{|}_{\begin{array}{c} s=s_{d}\\ h=h_{d} \end{array}}\;(\text{s}-{\text{s}}_{\text{d}})+\frac{\delta \text{f}}{\delta \text{h}}{|}_{\begin{array}{c} s=s_{d}\\ h=h_{d} \end{array}}\;(\text{h}-{\text{h}}_{\text{d}})$$

The linear error model of the robot is obtained by subtracting Equation (11) from (14):


(15) $$\begin{array}{l} \overset{•}{\tilde{\text{s}}}={\text{E}}_{\text{t}}\tilde{\text{s}}+{\text{F}}_{\text{t}}\tilde{\text{h}} \end{array}$$

where $\dot{\tilde{\text{s}}}={\dot{\text{s}}}_{d}-\dot{\text{s}},\tilde{\text{s}}={\text{s}}_{\text{d}}-\text{s},\tilde{\text{h}}={\text{h}}_{\text{d}}-\text{h},{\text{E}}_{t}=\frac{\delta \text{f}}{\delta \text{s}}{\left|{}_{\begin{array}{c} s=s_{d}\\ h=h_{d} \end{array}}\;,{\text{F}}_{\text{t}}=\frac{\delta \text{f}}{\delta \text{h}}\right|}_{\begin{array}{c} s=s_{d}\\ h=h_{d} \end{array}}$.

### Prediction model

To be adopted by the MPC controller, Equation (15) is discretized as follows:


(16) $$\begin{array}{l} \tilde{\text{s}}\left(m+1\right)={\text{E}}_{t}\tilde{\text{s}}\left(m\right)+{\text{F}}_{t}\tilde{\text{w}}\left(m\right) \end{array}$$

In order to compute system outputs in the future time horizon, Equation (16) is converted to the state space form $\xi \left(m|t\right)=\left[\begin{array}{c} \tilde{\text{s}}\left(m|t\right)\\ \tilde{\text{w}}\left(m-1|t\right) \end{array}\right]$, and the new state space is as follows:


(17) $$\begin{array}{l} \text{ξ}\left(m+1|t\right)={\tilde{\text{E}}}_{t}\text{ξ}\left(m|t\right)+{\tilde{\text{F}}}_{t}\text{Δ}\text{w}\left(m|t\right) \end{array}$$

(18) $$\begin{array}{l} \text{s}\left(m|t\right)={\tilde{\text{G}}}_{t}\text{ξ}\left(m|t\right) \end{array}$$

where ${\tilde{\text{E}}}_{t}=\left[\begin{array}{cc} {\text{E}}_{t} & {\text{F}}_{t}\\ 0_{i\times j} & {\text{I}}_{i} \end{array}\right],{\tilde{\text{F}}}_{t}=\left[\begin{array}{c} {\text{F}}_{t}\\ {\text{I}}_{i} \end{array}\right],{\tilde{\text{G}}}_{t}=[\begin{array}{cc} {\text{G}}_{t} & 0] \end{array}$ *j* = 5 and *i* = 2, ${\text{I}}_{i}=\left[\begin{array}{cc} 1 & 0\\ 0 & 1 \end{array}\right]$. Thus, the predictive output is described as follows:


(19) $$\begin{array}{l} \text{Z}\left(t\right)={\text{Ψ}}_{t}\text{ξ}\left(t|t\right)+{\text{Γ}}_{t}\text{Δ}\text{W}\left(t\right) \end{array}$$

where $\text{Z}(t)=\left[\begin{array}{c} s(t+1|t)\\ \vdots \\ s(t+M_{q}|t) \end{array}\right],\text{ψ}(t)=\left[\begin{array}{c} {\tilde{\text{G}}}_{t}{\tilde{\text{E}}}_{t}\\ \vdots \\ {\tilde{\text{G}}}_{t}{\tilde{\text{E}}}_{t}{}^{M_{q}} \end{array}\right],\Delta W=\;\left[\begin{array}{c} \Delta w(t|t)\\ \vdots \\ \Delta w(t+M_{d}|t) \end{array}\right],\;{\text{Γ}}_{t}=\left[\begin{array}{cccc} {\tilde{\text{G}}}_{t}{\tilde{\text{F}}}_{t} & 0 & 0 & 0\\ {\tilde{\text{G}}}_{t}{\tilde{\text{E}}}_{t}{\tilde{\text{F}}}_{t} & {\tilde{\text{G}}}_{t}\tilde{\text{F}} & 0 & 0\\ \vdots & \vdots & \ddots & \vdots \\ {\tilde{\text{G}}}_{t}{\tilde{\text{E}}}_{t}{}^{M_{q}-1}\tilde{\text{F}} & {\tilde{\text{G}}}_{t}{\tilde{\text{E}}}_{t}{}^{M_{q}-2}{\tilde{\text{F}}}_{t} & \cdots & {\tilde{\text{G}}}_{t}{\tilde{\text{E}}}_{t}{}^{M_{q}-M_{d}-1}{\tilde{\text{F}}}_{t} \end{array}\right].$

*M*_*q*_ and *M*_*d*_ are the predictive domain and control domain, respectively.

### QPSO rolling optimization

#### Objective function and constraints

By minimizing the deviation between the reference trajectory and the current trajectory, the CDCR achieves the optimal trajectory that tracks the desired path. The excessive tracking errors are penalized by the objective function.


(20) $$\begin{array}{l} \text{F}(m)=\sum_{j=1}^{M_{q}}{\left\|\text{s}(m+j|t)-{\text{s}}_{ref}(m+j|t)\right\|}_{\text{B}}^{2}\\ +\sum_{j=1}^{M_{d}-1}{\left\|\text{Δ}\text{w}(m+j|t)\right\|}_{\text{C}}^{2}+\rho \varepsilon^{2} \end{array}$$

where ***B*** , ***C*** represent the state increment weighting matrix and the control increment weighting matrix, respectively, *m*+*j|t* is the state quantity of *m*+*j* step at time *t*, Δ***w*** is the control increment, ρ represents weight coefficient, ε stands for relaxation factor.

The control quantity and control increment constraints need to be appended to avoid the sharp mutation of the speed when the servo controllers pull the cables, which are defined as follows:


(21) $$\begin{array}{l} {\text{w}}_{\min}\left(t+m\right)\leq \text{w}\left(t+m\right)\leq {\text{w}}_{\max}\left(t+m\right) \end{array}$$

(22) $$\begin{array}{l} \text{Δ}{\text{w}}_{\min}\left(t+m\right)\leq \text{Δ}\text{w}\left(t+m\right)\leq \text{Δ}{\text{w}}_{\max}\left(t+m\right) \end{array}$$

where *m* = 0, 1, …,*M*_*d*_-1.

In addition, the movement of the robot should be limited within its feasible region, which is defined as follows:


(23) $$\begin{array}{l} {\text{s}}_{\min}\left(t+m\right)\leq \text{s}\left(t+m\right)\leq {\text{s}}_{\max}\left(t+m\right) \end{array}$$

where *m* = 0, 1, …, *M*_*q*_-1.

Therefore, the objective function is as follows:


(24) $$\begin{array}{l} \text{F}\left(\text{ξ}\left(t\right),\text{w}\left(t-1\right),\text{Δ}\text{W}\left(t\right)\right)=\text{Δ}\text{W}\left(t\right){\text{H}}_{t}\text{Δ}\text{W}{\left(t\right)}^{\text{T}}+{\text{L}}_{t}\text{Δ}\text{W}{\left(t\right)}^{\text{T}} \end{array}$$

where the positive definite matrix $H_{t}=\left[\begin{array}{cc} \Gamma_{\text{t}}^{\text{T}}\text{B}\Gamma_{t}+\text{C} & 0\\ 0 & \rho \end{array}\right]$ is used to punish the control change rate. ${\text{L}}_{t}=\left[\begin{array}{cc} 2\text{D}{\left(t\right)}^{\text{T}}\text{B}\Gamma_{t} & 0 \end{array}\right],$ where *D*(*t*) is the tracking error.

Sort out Equations (21)–(24), the optimization process under constraints that need to be solve by QPSO can be presented as follows:


(25) $$\begin{array}{l} \;\underset{\text{Δ}\text{W}\left(t\right)}{\min}\text{Δ}\text{W}\left(t\right){\text{H}}_{t}\text{Δ}s\text{W}{\left(t\right)}^{\text{T}}+{\text{L}}_{t}\text{Δ}\text{W}{\left(t\right)}^{\text{T}}\\ \;\text{Δ}{\text{W}}_{\min}\leq \text{Δ}{\text{W}}_{t}\leq \text{Δ}{\text{W}}_{\max}\\ s.t.\;{\text{W}}_{\min}\leq \text{Δ}{\text{W}}_{t}+{\text{W}}_{t}\leq {\text{W}}_{\max}\\ \;{\text{Z}}_{\min}\leq {\text{Z}}_{\text{t}}\leq {\text{Z}}_{\max} \end{array}$$

#### QPSO algorithm

Classical PSO algorithm only allows the optimal particles to appear in a limited space for lack of randomness, which can lead to local minima. In contrast, QPSO allows particles to appear anywhere in the whole space with a certain probability, which improves the performance of optimization. Besides, there is only one parameter of QPSO need to be tuned, which makes it easy to be implemented. In the QPSO model, particles update their positions in accordance with the following equations:


(26) $$\begin{array}{l} mbest_{y}\left(t\right)=\overset{N}{\underset{x=1}{\sum }}\frac{P_{xy}\left(t\right)}{\text{N}}=\left(\overset{N}{\underset{x=1}{\sum }}\frac{P_{x1}\left(t\right)}{\text{N}},\ldots ,\overset{N}{\underset{x=1}{\sum }}\frac{P_{xe}\left(t\right)}{\text{N}}\right) \end{array}$$

(27) $$\begin{array}{l} PP_{xy}\left(t\right)=\sigma_{y}\left(t\right)P_{xy}\left(t\right)+\left[1-\sigma_{y}\left(t\right)\right]P_{gy}\left(t\right) \end{array}$$

(28) $$\begin{array}{l} I_{xy}\left(t+1\right)=PP_{xy}\left(t\right)\pm \eta |mbest_{y}\left(t\right)-I_{xy}\left(t\right)|\ln\;\left[\frac{1}{ra_{xy}\left(t\right)}\right] \end{array}$$

where *mbest*(*t*) represents the average of all the particles' optimal positions, vector *P*_*x*_(*t*) = (*P*__*x*_1_(*t*), *P*__*x*_2_(*t*), …, *P*_*xe*_(*t*)) represents the optimal individual position of particles, vector *P*_*g*_(*t*) = (*P*__*g*_1_(*t*), *P*__*g*_2_(*t*), …, *P*_*gy*_(*t*)) represents the global best position of particles, vector *I*_*xy*_(*t*) = (*I*__*x*_1_(*t*), *I*__*x*_2_(*t*), …, *I*_*xe*_(*t*)) represents the particle position, *PP*_*xy*_(*t*) represents the stochastic point between *P*_*x*_(*t*) and *P*_*g*_(*t*), *N is* the total number of particles and *e* is the dimension of particles. Random parameters σ and *ra*(*t*) are distributed uniformly in [0, 1]. η represents shrinkage and expansion coefficient, which is used to adjust the convergence speed of the controller. The QPSO algorithm is described as follows:

(1) Initialize the particle swarm, where *I*_*xy*_ is a random value.
(2) Calculate *mbest*(*t*) by Equation (26).
(3) Calculate the loss function value *F*(*I*_*xy*_(*t*+1)) according to Equation (25). Update *P*_*x*_(*t*) and *P*_*g*_(*t*).
(4) Update the new position *I*_*xy*_(*t*+1) of all particles according to Equation (28).
(5) Let *t* = *t*+1, and skip back to step 2 until the algorithm reaches the maximum number of iterations.

By derivatizing Equation (2), the tensile speed of the driving cables is computed as follows:


(29) $$\begin{array}{l} \overset{.}{\text{Δ}l_{i}}=d\left(\overset{.}{\theta }\cos\left(\text{Γ}-\alpha \right)-\theta \overset{.}{\varphi }\sin\left(\text{Γ}-\alpha \right)\right) \end{array}$$

where $\overset{.}{\text{Δ}l_{i}}$ stands for the driving speed of *i*-th cable, Γ = (*n*- 1)π/2 is the division angle.

## Simulation results

**Figure 4:**
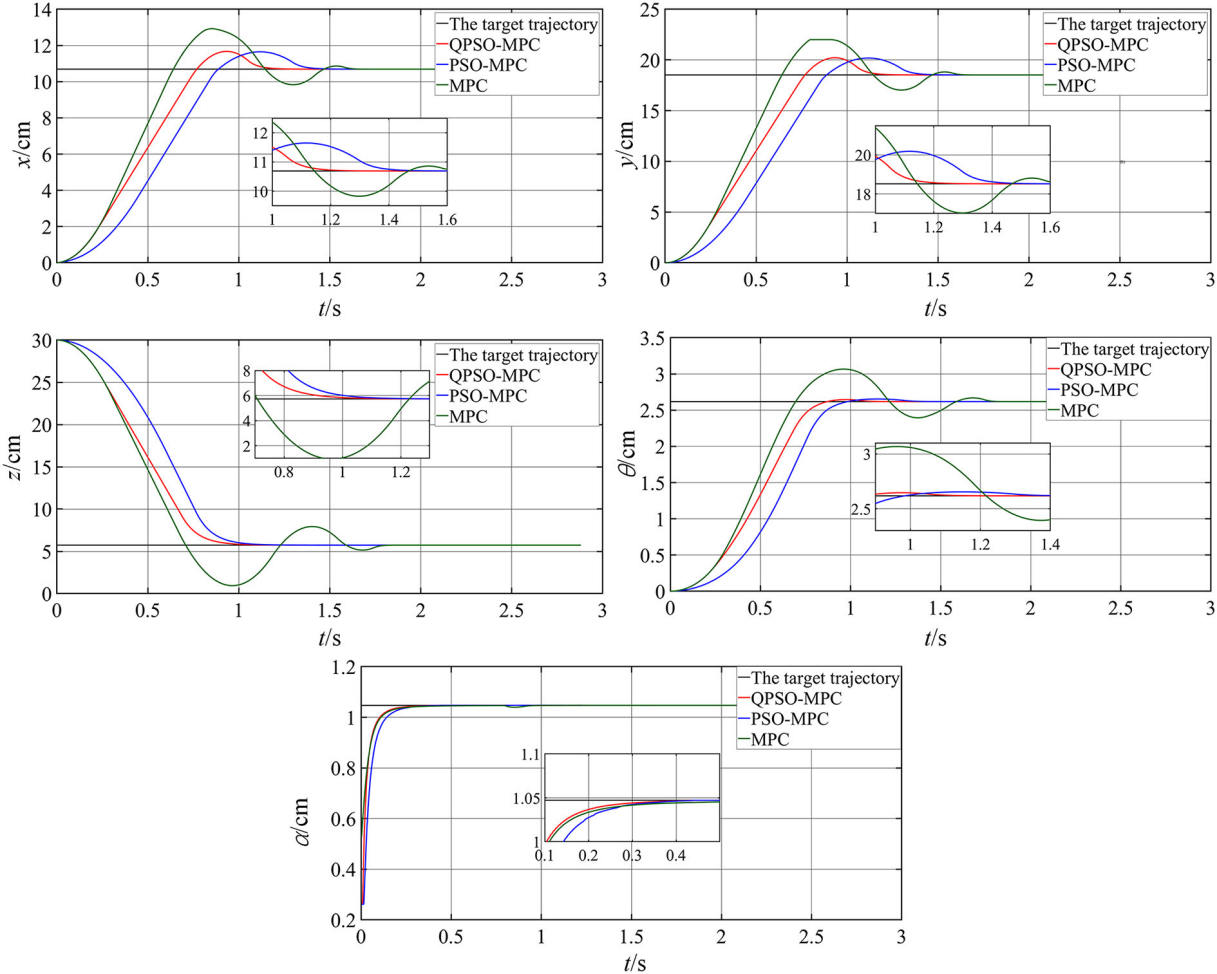
Simulation results of setpoint tracking.

A set of simulations have been made to evaluate the effectiveness of the proposed QPSO-MPC controller in comparison with a conventional MPC controller and a PSO-MPC controller. For simulation studies, the initial positions of the robot are set to the natural relaxation state, where the initial position vector is set as [0, 0, *l*, 0, 0]^T^ and the flexible support length *l* is 30 cm. The parameters are set as Table 1, where *Ni*_max_ means the maximum number of iterations. In the first simulation, the static performances of the three controllers are verified by the setpoint trajectory tracking. The static target position of the robot is set as [10.69, 18.52, 5.73, 5π/6, π/3]^T^ and the sampling period is 0.05 s. The simulation results of the three controllers are shown in Figure 4.

**Table 1:** The control parameters for the three controllers.

**Controller**	***M_*q*_***	***M_*d*_***	***N***	***Ni*_max_**
QPSO-MPC	20	1	50	50
PSO-MPC	20	1	50	50
MPC	20	1	/	/

It can be seen that all the three controllers track the desired position accurately within a certain time period and make the robot deformed into the desired shape. However, the QPSO-MPC controller yields smaller overshoots and more accurate tracking performance.

In the second simulation, the dynamic trajectory tracking performance is evaluated by tracking the circular-shaped trajectory, which is defined as follows:


(30) $$\left\{ \begin{array}{l} x=l(1\;-\text{ cos}\theta \text{)cos}\alpha /\theta \\ y=l(1\;-\text{ cos}\theta \text{)sin}\alpha /\theta \\ z=l\text{sin}\theta /\theta \\ \theta =\pi /2\\ \alpha =t\;t\in [0,2\pi ] \end{array}\right.$$

**Figure 5:**
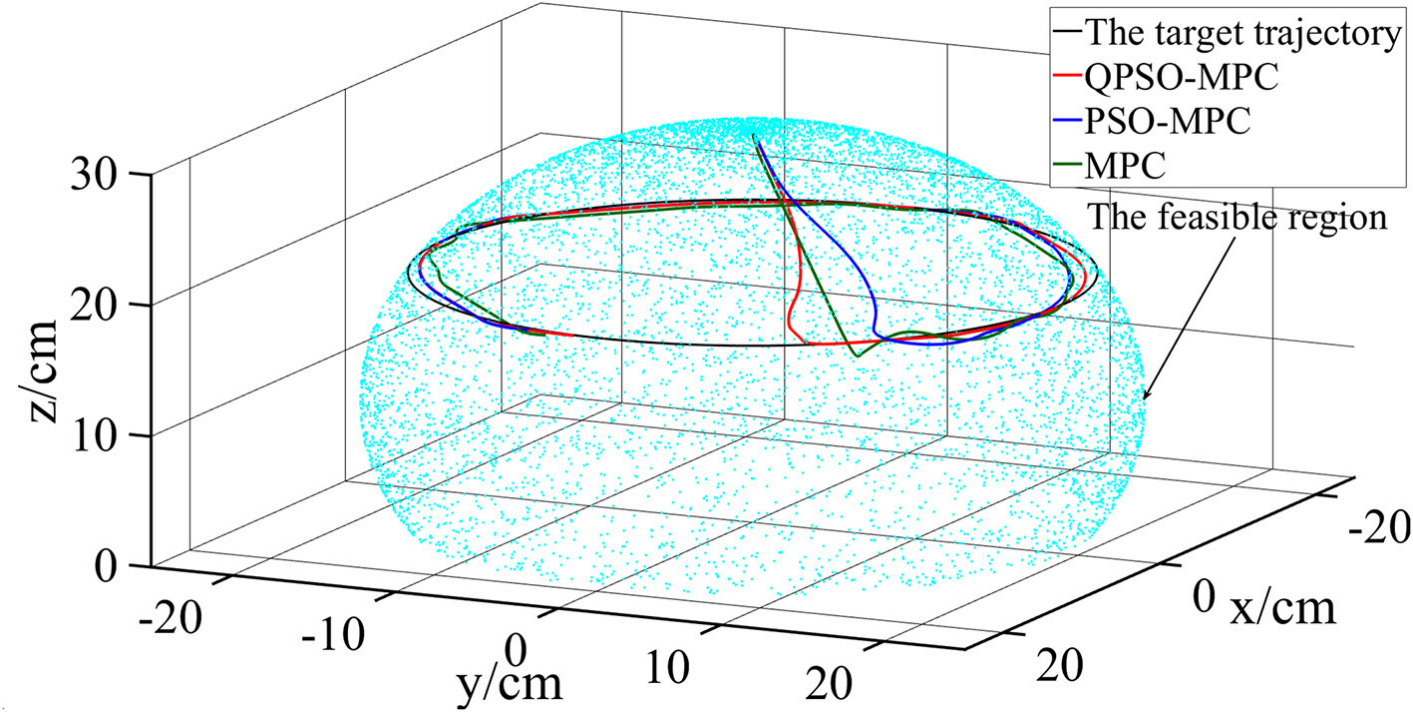
Simulation results of circular trajectory tracking.

**Figure 6:**
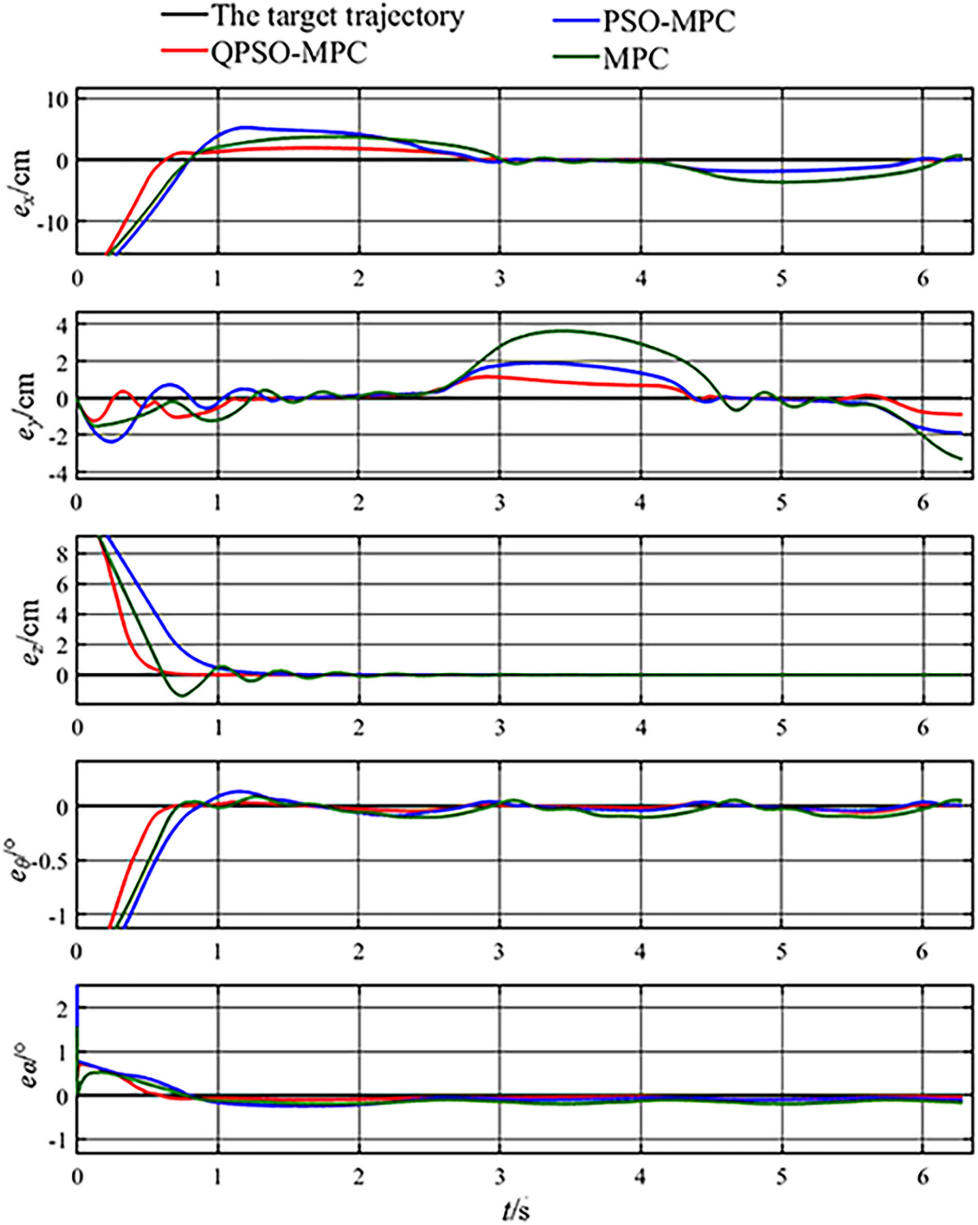
Tracking errors of circular trajectory tracking.

The simulation results of circular trajectory tracking are shown in Figure 5. The tracking errors of circular trajectory tracking are given in Figure 6. The tracking errors of the QPSO-MPC are smaller than those of other controllers. The tracking errors of QPSO-MPC, PSO-MPC and MPC controllers are 2.64, 3.73, and 4.16 cm, respectively. The response time of QPSO-MPC, PSO-MPC and MPC controllers are 3.0, 3.1, and 3.5 s, respectively. The simulation results verify the effectiveness of the control sequences generated by the QPSO-MPC in suppressing the system overshoot, reducing oscillation, and improving convergence.

## Experiment results

**Figure 7:**
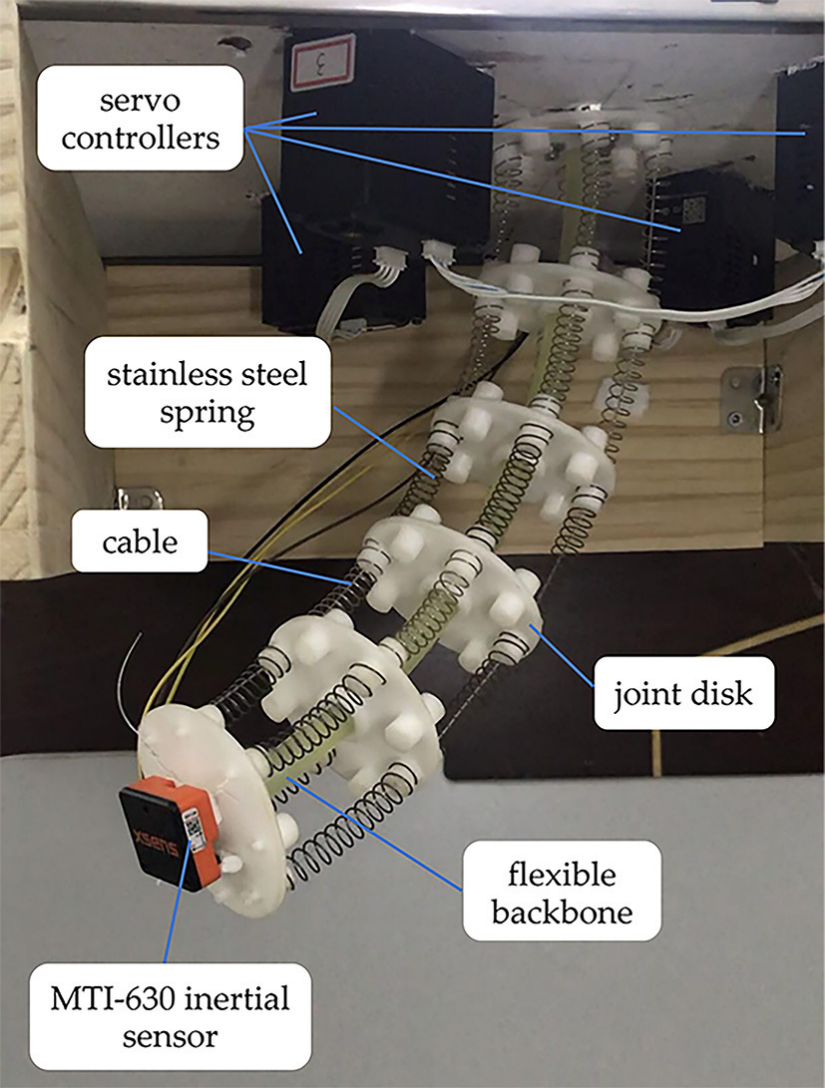
Experimental platform of the CDCR.

Experimental implementations are utilized to verify the trajectory tracking effectiveness of the QPSO-MPC controller in comparison with the PSO-MPC and the conventional MPC controller. The CDCR is shown in Figure 7, which consists of a flexible backbone (diameter, 1 cm; length, 30 cm), a MTI-630 inertial sensor, four anti-winding driving cables (diameter, 0.1 cm), four servo motors with reducers (servo motors type, QDD Plus-NU80-6; reduction ratio, 6:1; maximum torque, 6 N·m; rated full-load speed, 200 rpm), six joint disks (diameter, 4 cm; distance between joint disks, 6 cm) and twenty stainless steel springs. The servo motors are used in Position Mode. The flexible backbone provides bending stiffness for the robot. Each joint disk, mounted on the flexible backbone, has eight uniformly distributed cable holes.

The stainless steel springs (diameter, 1 cm) are used to maintain the external contour of the robot. The servo controllers, using CAN bus communication, are evenly distributed on the operating chassis. The MTI-630 inertial sensor is an industrial inertial measurement unit (IMU) and the direction error is <0.5°, which can produce an accurate measurement of the attitude and position of the robot end-effector. In order to ensure the trajectory tracking performance of the CDCR, the maximal load that the end-effector can support is 300 g. A higher maximal load of the CDCR can be achieved by increasing backbone strength, spring stiffness, cable strength, and motor power. The double-integration of the acceleration measured from the MTI-630 sensor is processed by the Extended Kalman Filter, which can reduce the estimated error of position and provide reliable motion data for trajectory tracking. Besides, when the output of the servo motors is zero, the velocity should also be zero, which can be used for the Extended Kalman Filter to reduce zero-shift effect.

In this paper, the tracking performances of QPSO-MPC, PSO-MPC and MPC controllers are assessed with respect to three typical trajectory tracking tasks: the cardioid curve, the polyline curve and the spiral curve, which can be presented as (31), (32), and (33), respectively.


(31) $$\{\begin{array}{l} x=l(\text{1-cos}\theta )\text{cos}\alpha \text{/}\theta \\ y=l(\text{1-cos}\theta )\text{sin}\alpha \text{/}\theta \\ z=l\sin\theta /\theta \\ \theta =\{\begin{array}{l} \pi /6+2\pi \times t/3t\in (0,\pi ]\\ 3\pi /2-2\pi \times t/3t\in (\pi ,2\pi ] \end{array}\\ \alpha =t\;t\in (0,2\pi ] \end{array}$$

(32) $$\{\begin{array}{l} x=l(\text{1-cos}\theta )\text{cos}\alpha \text{/}\theta \\ y=l(\text{1-cos}\theta )\text{sin}\alpha \text{/}\theta \\ z=l\sin\theta /\theta \\ \theta \text{​}=\text{​}\{\begin{array}{ll} \left|\text{​}1.5\text{​}\times l\text{sin}(t)\right| & t\in [\pi /4,3\pi /4]\cup [5\pi /4,7\pi /4]\;\\ \left|1.5\times l\text{cos}(t)\right| & other \end{array}\\ \alpha =t\;t\in (0,2\pi ] \end{array}$$

(33) $$\left\{ \begin{array}{l} x=l(\text{1-cos}\theta )\text{cos}\alpha \text{/}\theta \\ y=l(\text{1-cos}\theta )\text{sin}\alpha \text{/}\theta \\ z=l\text{sin}\theta \text{/}\theta \\ \theta =\pi t/4+\pi /3t\in (0,2\pi ]\;\\ \;\alpha =2tt\in (0,2\pi ] \end{array}\right.$$

**Figure 8:**
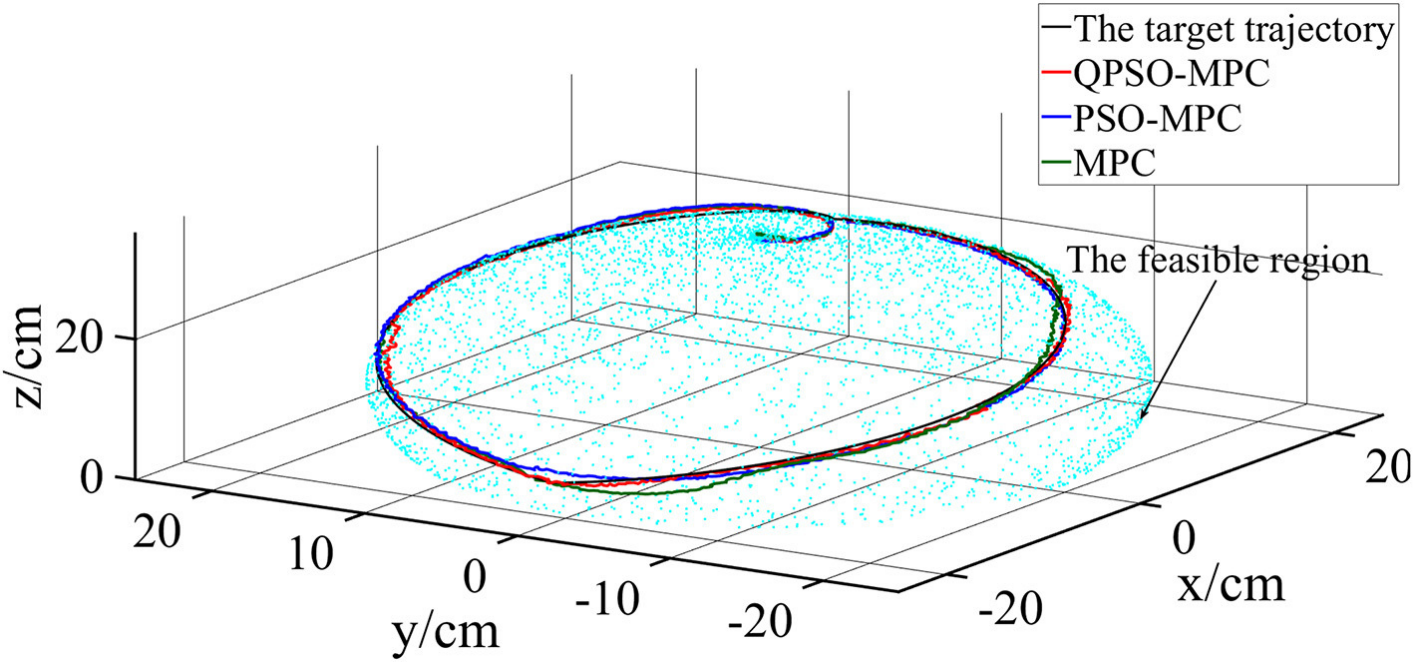
Experiment results of the cardioid trajectory tracking.

**Figure 9:**
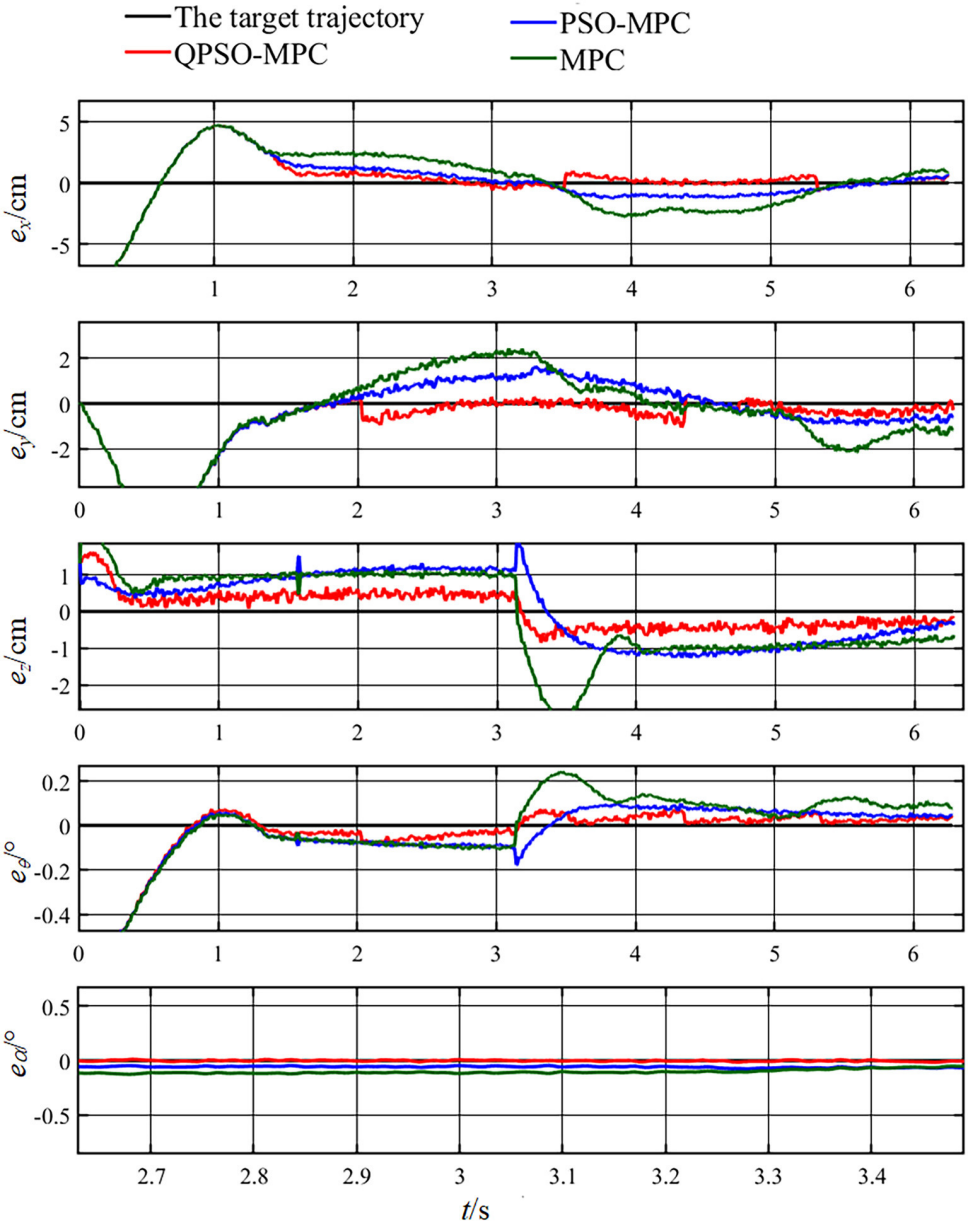
Tracking errors of the cardioid trajectory tracking.

**Figure 10:**
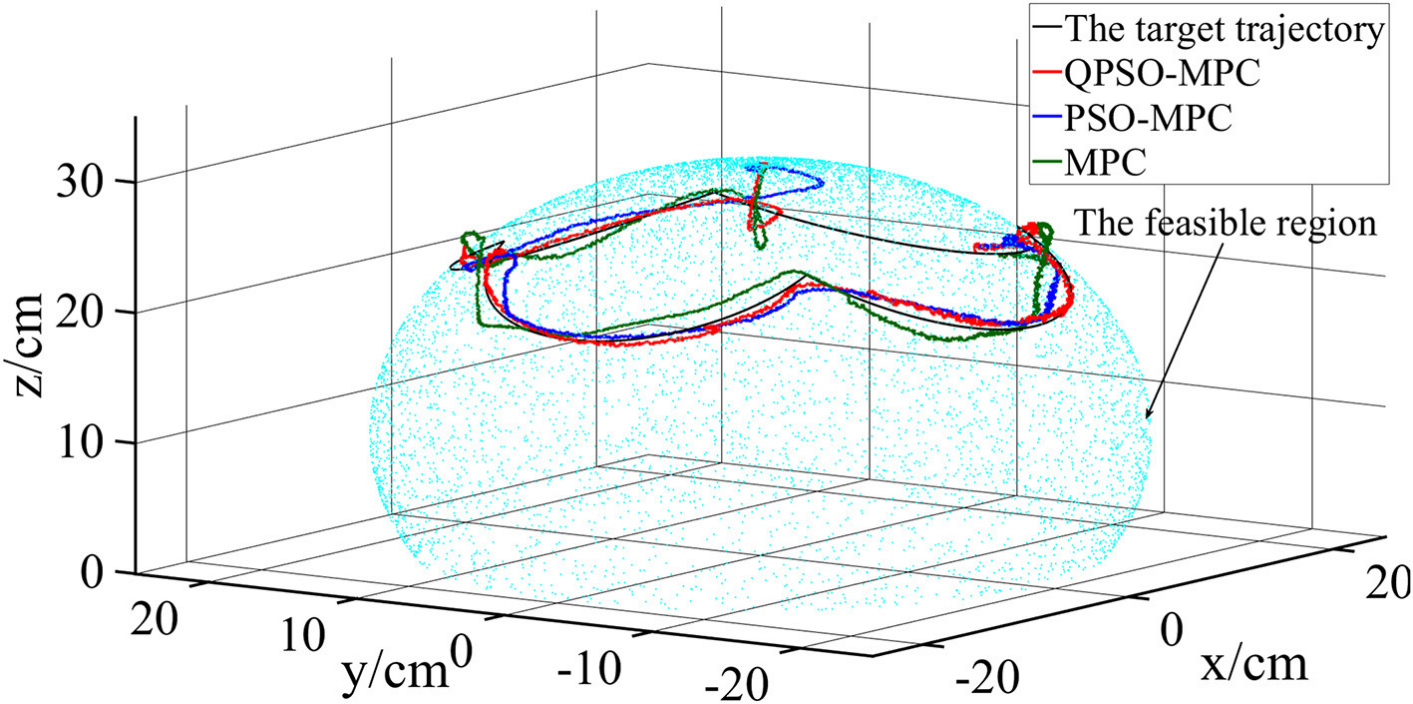
Experiment results of the polyline trajectory tracking.

**Figure 11:**
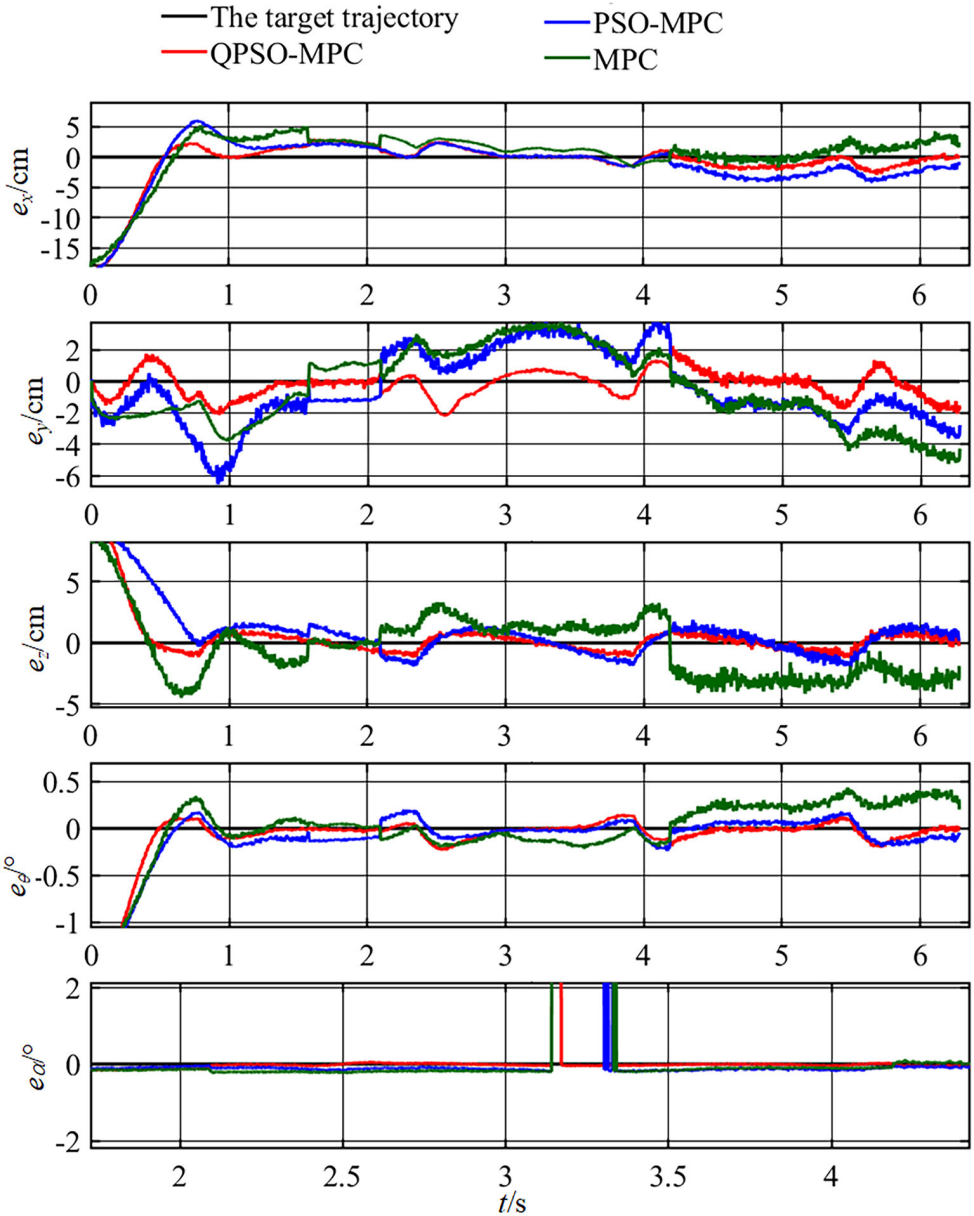
Tracking errors of the polyline trajectory tracking.

**Figure 12:**
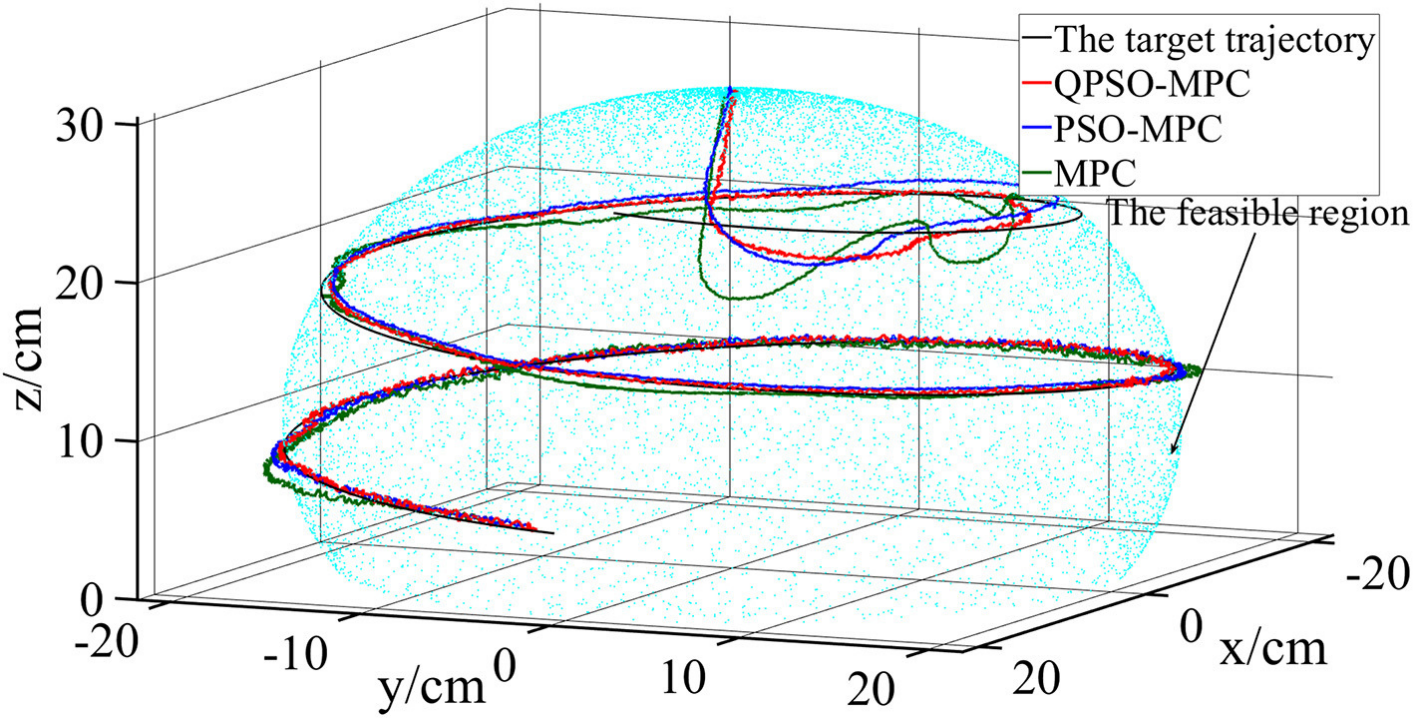
Experiment results of the spiral trajectory tracking.

**Figure 13:**
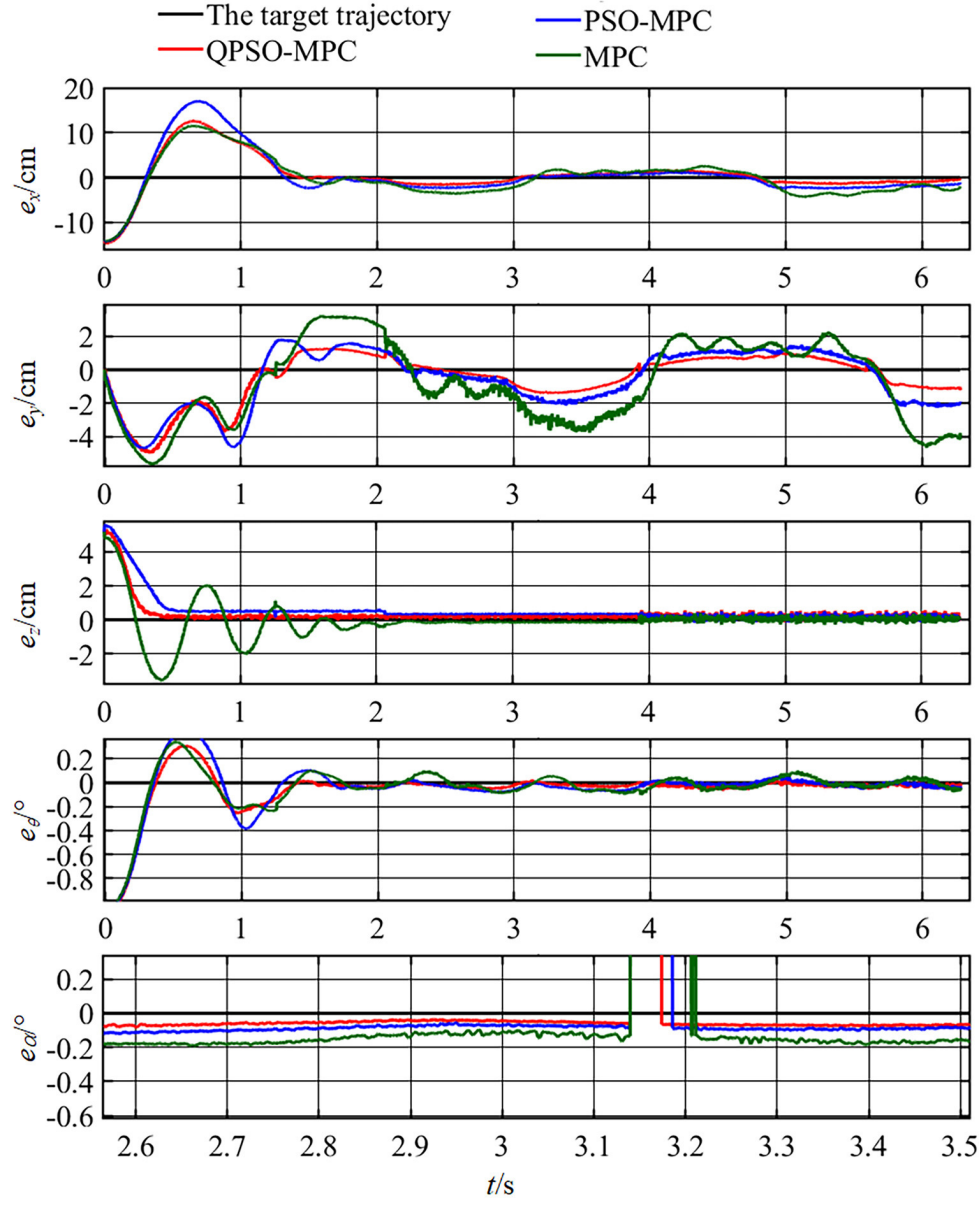
Tracking errors of the spiral trajectory tracking.

The control parameters of all the controllers for the experiments are set as follows in Table 1. The sampling periods of the three tasks are 0.05, 0.02, and 0.01 s, respectively. The tracking results and tracking error curves of the cardioid trajectory are shown in Figures 8, 9, respectively. The tracking results and tracking error of the polyline trajectory are shown in Figures 10, 11, respectively. The tracking results and tracking error of the spiral trajectory are shown in Figures 12, 13, respectively. It is shown that all the controllers could control the robot to move along the reference trajectories within a certain time. However, the QPSO-MPC controller fits the reference trajectory best in all the three tracking tasks.

The tracking errors are quantified as the mean value of the Euclidean distance between the real position and the target trajectory, which are shown in Table 2. In each task, the QPSO-MPC controller achieves the lowest tracking errors. The average tracking errors of QPSO-MPC, PSO-MPC and MPC controllers in all the three tasks are 2.61, 3.25, and 3.74 cm respectively, and the standard deviations are 0.44, 0.48, and 0.62 cm, respectively. Compared with the PSO-MPC and the MPC controllers, the average tracking errors of the QPSO-MPC is reduced by 24.52 and 43.30%, respectively, which further proves the tracking effectiveness of QPSO-MPC outperforms the other controllers. The experiments show that QPSO-MPC controller achieves accurate and stable tracking performance in complex trajectory tracking tasks. The proposed QPSO-MPC controller achieves substantial improved performance by comparing with MPC and PSO-MPC controllers because it ensures the system get a global optimal output at every moment.

**Table 2:** The tracking errors of the experiments.

**Controller**	**Error (cm)**			**Average** **error (cm)**	**Standard deviation (cm)**
	**Task 1**	**Task 2**	**Task 3**		
QPSO-MPC	2.19	2.57	3.07	2.61	0.44
PSO-MPC	2.79	3.22	3.74	3.25	0.48
MPC	3.05	3.91	4.26	3.74	0.62

## Conclusions

In this paper, a QPSO-MPC is applied to trajectory tracking tasks of CDCRs. The kinematic model of the CDCR is built based on the piecewise constant curvature assumptions, which simplifies the mathematical model of the CDCR and reduces the computation of the control system. The QPSO is applied to the rolling optimization process of the MPC based controller, which guarantees stable and accurate trajectory tracking under constraints. The QPSO provides optimal control outputs of MPC to compensate various uncertainties. Moreover, the QPSO solves the local minima problem of PSO algorithm.

The effectiveness of the proposed QPSO-MPC in typical tracking tasks is demonstrated by both simulations and experiments. Compared with PSO-MPC and MPC controllers, the QPSO-MPC algorithm shows greatly improved performance in three typical tracking tasks. It has been proved that the QPSO-MPC controller is more suitable for controlling CDCRs to track complex trajectories. Future studies will focus on the extended applications of the QPSO-MPC algorithm for CDCRs with different structures, such as CDCRs with multi-backbone structures and CDCRs with nested backbone.

## Data availability statement

The raw data supporting the conclusions of this article will be made available by the authors, without undue reservation.

## Author contributions

QC and YQ conceived the study and put forward the methodology. YQ and GL performed the data collection and pre-processing, and reviewed and edited the manuscript. QC carried out the software for the experiments and wrote the first draft of the manuscript. All authors read and agreed to the published version of the manuscript.

## Funding

This research was funded by National Natural Science Foundation of China Grant Numbers 51975565 and 52127813, Science and Technology Innovation Plan of Shanghai Science and Technology Commission Grant Number 20DZ1206700, and Natural Science Foundation of Liaoning Province Grant Number 2020-KF-22-12.

## Conflict of interest

The authors declare that the research was conducted in the absence of any commercial or financial relationships that could be construed as a potential conflict of interest.

## Publisher's note

All claims expressed in this article are solely those of the authors and do not necessarily represent those of their affiliated organizations, or those of the publisher, the editors and the reviewers. Any product that may be evaluated in this article, or claim that may be made by its manufacturer, is not guaranteed or endorsed by the publisher.
